# Efficient CRISPR/Cas9-Mediated Knockout of an Endogenous *PHYTOENE DESATURASE* Gene in T1 Progeny of Apomictic *Hieracium* Enables New Strategies for Apomixis Gene Identification

**DOI:** 10.3390/genes11091064

**Published:** 2020-09-10

**Authors:** Sam W. Henderson, Steven T. Henderson, Marc Goetz, Anna M. G. Koltunow

**Affiliations:** Commonwealth Scientific and Industrial Research Organisation (CSIRO) Agriculture and Food, Glen Osmond, SA 5064, Australia; steven.henderson@csiro.au (S.T.H.); marc.goetz@csiro.au (M.G.)

**Keywords:** *Hieracium piloselloides*, apomixis, CRISPR/Cas9, *PHYTOENE DESATURASE* (*PDS*), amplicon sequencing, genome editing, tissue culture

## Abstract

Most *Hieracium* subgenus *Pilosella* species are self-incompatible. Some undergo facultative apomixis where most seeds form asexually with a maternal genotype. Most embryo sacs develop by mitosis, without meiosis and seeds form without fertilization. Apomixis is controlled by dominant loci where recombination is suppressed. Loci deletion by γ-irradiation results in reversion to sexual reproduction. Targeted mutagenesis of genes at identified loci would facilitate causal gene identification. In this study, the efficacy of CRISPR/Cas9 editing was examined in apomictic *Hieracium* by targeting mutations in the endogenous *PHYTOENE DESATURASE* (*PDS*) gene using *Agrobacterium*-mediated leaf disk transformation. In three experiments, the expected albino dwarf-lethal phenotype, characteristic of *PDS* knockout, was evident in 11% of T0 plants, 31.4% were sectorial albino chimeras, and the remainder were green. The chimeric plants flowered. Germinated T1 seeds derived from apomictic reproduction in two chimeric plants were phenotyped and sequenced to identify *PDS* gene edits. Up to 86% of seeds produced albino seedlings with complete *PDS* knockout. This was attributed to continuing Cas9-mediated editing in chimeric plants during apomictic seed formation preventing *Cas9* segregation from the *PDS* target. This successful demonstration of efficient CRISPR/Cas9 gene editing in apomictic *Hieracium*, enabled development of the discussed strategies for future identification of causal apomixis genes.

## 1. Introduction

**Figure 1:**
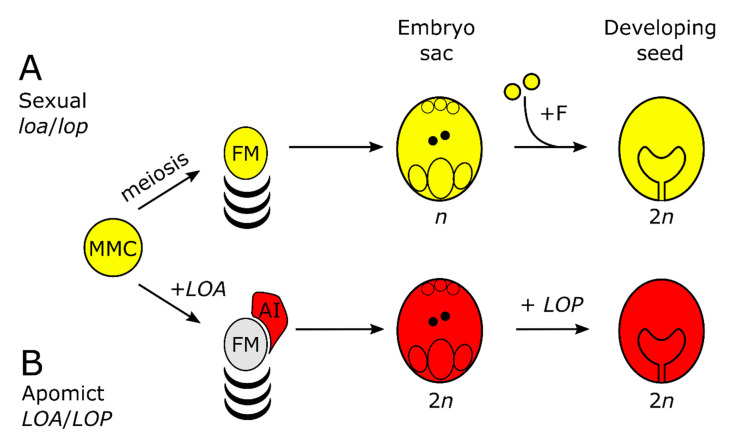
Simplified schematic of sexual and apomictic reproduction in *Hieracium* subgenus *Pilosella* (**A**) The sequence of events in sexual *Hieracium pilosella* indicated in yellow. MMC indicates megaspore mother cell, +F = fertilization, FM = functional megaspore; (**B**) events of apomixis (red) in *Hieracium praealtum* (R35) and events controlled by activity of the *LOSS OF APOSPORY* (*LOA*) and *LOSS OF PARTHENOGENESIS* (*LOP*) loci. AI = aposporous initial cell.

Increasing seed yields in major crops requires new insights into the function of genes that regulate plant reproduction. Plants typically form seeds via a sexual pathway requiring meiosis to form male and female gametes and fertilization to initiate seed formation (Figure 1A). A number of non-agronomic plants can also form seed asexually via apomixis. Apomixis is typically a dominant trait. However, most apomicts are facultative, meaning the sexual pathway remains intact in some ovules. During apomixis, genetically identical seeds of a maternal genotype form because meiosis is avoided during female gametophyte or embryo sac formation, and fertilization is not required for embryo formation [1,2,3]. Harnessing apomixis in plant breeding would have significant benefits for seed production and accelerate the delivery of hybrid crops with improved traits [4]. Attempts to introgress apomixis into sexual seed crops from apomictic relatives, which are typically polyploid, have been unsuccessful [5]. Furthermore, the suppression of recombination around apomixis loci in most molecularly studied apomicts has consistently hindered positional cloning to identify apomixis genes [2]. To date, the only known gene identified from a gametophytic apomict is that conferring fertilization-independent embryogenesis (parthenogenesis) from *Pennisetum squamulatum* [6].

Targeted genomic deletions, together with mutations in genes at apomixis loci would facilitate identification of additional apomixis genes. However, CRISPR/Cas9-mediated genome editing, which takes advantage of the cellular DNA double-strand break repair pathways to generate small indels, targeted substitutions and multiplex genome modifications [7], has not yet been reported in a gametophytic apomict. The application of CRISPR has been demonstrated to alter gene function in polyembryonic *Citrus* [8,9], wherein nucellar embryos form via fertilization-independent sporophytic apomixis [2]. However, those studies did not focus on identification of causal genes for apomixis utilizing this technology.

*Hieracium* subgenus *Pilosella* species, members of the Asteraceae, have been developed as a eudicot model for the molecular analysis of gametophytic apomixis. Sexual and apomictic species are self-incompatible, therefore seeds formed in the absence of fertilization are easily identified. In characterized apomicts, more than 95% of seed is formed via the facultative apomictic pathway [10], which is dependent on the initiation of the sexual pathway (Figure 1B). During apomixis, somatic aposporous initial (AI) cells differentiate near cells undergoing meiosis, the sexual pathway terminates, and the AI cells undergo mitosis forming the (aposporous) embryo sac within which embryo and endosperm formation are fertilization-independent [11]. In apomictic *Hieracium praealtum* (R35) and *Hieracium piloselloides* (D36) the dominant, *LOSS OF APOMEIOSIS* (*LOA*) locus regulates AI cell formation, suppression of the sexual pathway and aposporous embryo sac formation. In R35 the dominant *LOSS OF PARTHENOGENESIS* (*LOP*; Figure 1B) locus controls fertilization-independent embryogenesis and endosperm formation [11,12]. An additional locus has been identified in D36 termed *AutE* which enables fertilization-independent endosperm formation [13,14].

Recombination is suppressed at identified apomixis loci. For example, the *LOA* locus is located near the distal end of a single chromosome and is surrounded by extensive repeats and transposons [15]. This is also a feature of the apomixis carrying chromosome in monocots *P. squamulatum* and *Cenchrus ciliaris* [16,17]. Despite these genetic features of apomicts, extensive genome and transcriptome resources have been developed for apomictic *Hieracium* [18,19], and the interrogation of *Hieracium* cell-type-specific transcriptomes has identified candidate genes and pathways for apomixis that require functional testing [19,20,21]. Chromosome walking has enabled the identification of additional genes at both the *LOA* and *LOP* loci [15,22]. Thus, it would be timely to develop rapid new methods to test candidate apomixis gene function.

Interestingly, γ irradiation-induced deletions of the *LOA* and *LOP* loci in apomictic *H. praealtum* (R35) have shown phenotypic reversion to the sexual mode of reproduction. This indicates that sexual reproduction is the default reproductive mode [11,12]. Targeted deletions in genomic regions and genes linked to and within at *LOA* and *LOP* loci from chromosome walking would conceivably enable the functional characterization of apomixis genes. Importantly, unlike many apomict model species, the characterized sexual and apomictic subgenus *Pilosella* species can be efficiently transformed using *Agrobacterium* mediated leaf disc transformation [23].

CRISPR/Cas9 gene editing has been assessed in a small number of sexual Asteraceae species. The thermoinhibition gene *NCED4* was successfully edited using CRISPR/Cas9 in diploid, self-compatible lettuce (*Lactuca sativa*) with editing stability and *Cas9* segregation demonstrated in the next seed generation [24]. In both sexual diploid *Tragopogon porrifolius* and tetraploid *Tragopogon mirus,* a low-efficiency *Agrobacterium*-mediated transformation system was developed to examine CRISPR/Cas9 gene editing efficiency in T0 calli and regenerated shoots, however plants were not taken to the next generation [25]. In hexaploid self-incompatible *Chrysanthemum morifolium,* low-efficiency editing of an introduced fluorescent marker gene was observed [26]. CRISPR/Cas9 has also been evaluated in T0 plants of the rubber producing dandelion (*Taraxacum kok-saghyz*), a sexual relative of *Hieracium*, however due to self-incompatibility the analyses did not progress to the T1 generation [27]. The *Cas9* gene should be retained in progeny derived via the apomictic pathway and not segregate as typically occurs among sexually derived progeny. A knowledge of the efficiency of CRISPR/Cas9 gene editing is therefore required in apomictic *Hieracium* before embarking on large scale editing approaches to identify and characterize apomixis genes.

In this study, we targeted knockout of the endogenous phytoene desaturase (PDS) enzyme in tetraploid self-incompatible apomictic *Hieracium piloselloides*, D36 to assess CRISPR/Cas9 gene editing efficiency. PDS catalyzes the desaturation of phytoene to ζ-carotene during carotenoid biosynthesis and loss of *PDS* function in *Arabidopsis* results in a visible albino dwarf seedling-lethal phenotype [28]. *PDS* has been used to develop and evaluate CRISPR/Cas9 gene editing in crops including rice [29], cassava [30], wheat [31] and banana [32]. Due to the obvious albino phenotype, plants exhibiting complete *PDS* knockout in all tissues can be phenotypically identified, in addition to chimeric plants exhibiting albino sectors. *Agrobacterium*-mediated leaf disk transformation was used to introduce constructs targeting *Hieracium PDS* (*HPDS*). T0 regenerants and subsequent T1 progeny derived via apomictic reproduction in flowering chimeric T0 plants were analyzed. Editing events were assessed using amplicon deep sequencing. Albino seedlings increased in frequency in T1 progeny suggesting that editing continues during apomictic seed formation due to non-segregation of *Cas9*.

## 2. Materials and Methods

### 2.1. Plant Growth

*Hieracium piloselloides* (4x = 2n = 36) was maintained by vegetative micropropagation in vitro and grown in a glasshouse, as described previously [11].

### 2.2. Identification of an Hieracium Phytoene Desaturase (HPDS) Gene Ortholog and Confirmation of Expressed Leaf cDNA Sequence

The tetraploid *H. piloselloides*, D36 is closely related to diploid lettuce and shares the same base chromosome number (n = 9). Comparative genomic analysis between *Hieracium* species and lettuce has revealed partial macrosynteny for six linkage groups [33]. To identify lettuce PDS, a reciprocal best hit BLASTP search was performed with the *Arabidopsis* PDS amino acid sequence (NCBI accession AF360196) [28] against the lettuce genome. This revealed a 581 amino acid (aa) hypothetical protein (accession PLY83262.1) that showed 79% identity to *Arabidopsis* PDS. The amino acid sequence of lettuce PDS was then used in TBLASTN analysis against the *Hieracium* diploid plant D18 genomic DNA scaffold and tetraploid apomictic *Hieracium* D36 transcriptomes at http://hieracium.csiro.au [19]. This revealed a genomic DNA fragment (D18-gDNA-s59305) encoding a putative protein with 74.87% identity to lettuce PDS and additional transcript fragments (Figure S1A). Phylogenetic analysis confirmed the close relationship between lettuce and *Hieracium* PDS (Figure S2). The identified *HPDS* gene sequence was confirmed by isolating and sequencing the predicted gene from D36 leaf cDNA using the primers listed in Table S1. *Hieracium* leaf total RNA was isolated using the RNeasy Plant Mini Kit (Qiagen, Hilden, Germany). The RNA was treated with Turbo DNAse I (Ambion, Austin, TX, USA) to remove contaminating genomic DNA, ethanol precipitated and resuspended in nuclease-free water. First-strand cDNA was synthesized from 5 μL of RNA using SuperScript^®^ III First-Strand Synthesis SuperMix (Thermo Fisher Scientific, Waltham, MA, USA). The predicted full-length coding sequence of the *HPDS* gene was amplified by polymerase chain reaction (PCR) using 0.5-μM forward (SWH12) and reverse (SWH13) primers (Table S1), 1 μL of diluted (1:5) cDNA and 0.2 U of Phusion^®^ High-Fidelity DNA Polymerase (New England Biolabs, Ipswich, MA, USA) in a 20-μL reaction. The PCR product was purified using the Illustra GFX PCR DNA and Gel Band Purification Kit (GE Healthcare, Chicago, IL, USA) and Sanger sequenced.

### 2.3. Development of a CRISPR/Cas9 Gene Editing Construct Targeting Hieracium PDS

Three 20-nucleotide guide RNAs, beginning with a guanidine and binding directly upstream of an NGG protospacer adjacent motif (PAM), were designed using Geneious v. 11.0.4 (Biomatters, Auckland, New Zealand). Guides were analyzed for offsite targeting against the *Hieracium* diploid D18 genome sequence [19]. Complementary oligonucleotides encoding the three different sgRNAs (Table S1) were diluted to 4 μM in sterile water and self-annealed in a PCR cycler using 70 cycles of 95 °C for 35 s (−1 °C/cycle). Annealed oligos were ligated into the BsaI-HF site of the gateway entry vectors pEN-Comaira.1, pEN-Comaira.2 and pEN-Comaira.3. Multisite gateway was performed, using LR Clonase II plus (Thermo Fisher Scientific), to recombine the three U6: sgRNA constructs into pDE-Cas9 [34] and to produce a single expression construct containing three guides targeting *HPDS*.

### 2.4. In Vitro Cleavage Assay

The functional efficacy of the three sgRNAs to target *HPDS* was screened in vitro using the Guide-it Complete sgRNA Screening System (Takara Bio, Kusatsu, Shiga, Japan). The forward primers used for amplifying the templates for in vitro transcription of the sgRNAs are shown in Table S1. The substrate of the in vitro cleavage assays was a 2716 base pair (bp) PCR fragment of the *HPDS* gene generated using the primers SWH44 and SWH46 (Table S1). Briefly, the cleavage template (2.5 μL) was incubated with 50 ng of recombinant Cas9 protein and 250 ng of in vitro transcribed sgRNA in Cas9 Reaction Buffer at 37 °C for 1 h. Reactions were stopped by incubating at 80 °C for 5 minutes and resolved on a 1.5% agarose gel (Figure S3).

### 2.5. Plant Transformation

Constructs were electroporated into *Agrobacterium tumefaciens* strain AGL1. Plants were transformed using the leaf disk protocol of Bicknell and Borst, [23] with the exception that phosphinothricin (5 mg L^−1^) was used as the plant selectable marker instead of kanamycin for selecting positive transformants. To confirm the presence and integrity of the T-DNA in the phosphinothricin-resistant primary T0 transformants, the Extract-N-Amp Kit (Sigma-Aldrich, St. Louis, MO, USA) was used to amplify a 906 bp fragment of the *Cas9* coding sequence by PCR using primers SWH190 and SWH191 (Table S1), following manufacturer’s procedures.

### 2.6. Identification of Edits in HPDS Genes of Transformed Hieracium Plants by Sequencing

Genomic DNA was extracted from emerging leaves of young T0 plantlets using the Extract-N-Amp Kit (Sigma-Aldrich, MO, USA) for sequencing. Primers (SWH186 and SHW187), surrounding the predicted editing sites (Table S1), were used to amplify a 540 bp genomic DNA fragment of *HPDS* from a selection of transformed plants exhibiting green and white leaf phenotypes, with KAPA-HiFi hotstart high-fidelity polymerase (Roche, MA, USA). PCR fragments were Sanger sequenced at the Australian Genome Research Facility (AGRF). Sequence chromatograms with mixed spectra were analyzed using the Inference of CRISPR Edits (ICE) web tool at https://ice.synthego.com/.

For next generation sequencing (NGS), 18 independent T0 transformants and an untransformed D36 control, were analyzed. Genomic DNA was isolated from *Hieracium* leaf tissue using the DNeasy Plant Mini Kit (Qiagen), following the manufacturer’s protocol. Libraries were prepared using a two-step PCR protocol. For the first round PCR, primers were designed to generate two amplicons of ~200 bp each. The primers SWH200/201/202/203 spanned sgRNA1 and sgRNA2, while the primers SWH204/205/206/207 spanned sgRNA3 (Table S1). First-round PCR reactions contained 5 µM of each primer, 1 × KAPA HiFi Hotstart ReadyMix (Roche, Boston, MA, USA) and 10 ng of genomic DNA, in 20 µL. The PCR consisted of 35 cycles of 98 °C for 20 s, 60 °C for 15 s and 72 °C for 15 s. Amplicons were purified using Agencourt AMPure XP (Beckman Coulter, Brea, CA, USA). The second round PCR was carried out as the first round, except that Illumina Nextera i5 and i7 indexing primers were used, and 15 cycles were performed. Purified amplicons were pooled together, and the library was sequenced using a MiSeq (Illumina, Foster City, CA, USA) by the AGRF using the nano flow cell and 300 cycles. All next generation sequencing data were uploaded to the sequence read archive (SRA) at NCBI (Accession Number PRJNA636229).

Illumina paired-end reads were imported into Geneious (Biomatters) as fastq files. The sequences were paired, trimmed and then aligned to the amplicon reference sequence using Geneious Read Mapper with fast sensitivity. All sequence variants occurring at a frequency ≥1% from the reference amplicon were selected for analyses. These variants were further analyzed using CRISPResso [35] and CRISPResso2 [36].

### 2.7. Analyses of T1 Progeny Derived from Chimeric Apomictic T0 Transformed Plants

Seeds were harvested from mature plants and surface-sterilized with 12.5 g/L sodium hypochlorite and 0.05% (*v*/*v*) Triton X-100 for 5 min. Seeds were washed 4 times in sterile water and then plated onto media consisting of 0.5 × Murashige and Skoog Basal Salts, 3% (*w*/*v*) sucrose, pH 5.7 (KOH), 0.8% (*w*/*v*) plant agar and 1 × Gamborg’s vitamins (Sigma-Aldrich) without selection. Whole seedlings were harvested 10 days after germination. DNA was extracted from whole seedlings using the method described by Edwards et al. [37]. Edits in the T1 progeny were determined using amplicon deep sequencing on a MiSeq (Illumina) as described above.

## 3. Results

### 3.1. PDS Edited Apomictic Hieracium T0 Plants Show Dwarfism, Albinism and Chimeric Phenotypes in Vegetative and Floral Tissues

The 3.8 kb *HPDS* gene used as a phenotypic marker for gene editing in tetraploid apomictic *Hieracium* was predicted to contain 11 exons and encode a protein of 589 amino acids (Figure S1A). The CRISPR/Cas9 editing constructs designed to disrupt endogenous *HPDS* gene function, contained three sgRNAs targeting a 252 bp region within exon 10 (Figure S1A,B), which is similar to the region that was used to target *PDS* in cassava [30]. Expression of each guide was independently regulated by the *Arabidopsis* U6-26 promoters (Figure S1C). The completed *HPDS*-targeting CRISPR/Cas9 construct was introduced to cells of D36 leaf explants by *Agrobacterium*-mediated transformation.

**Figure 2:**
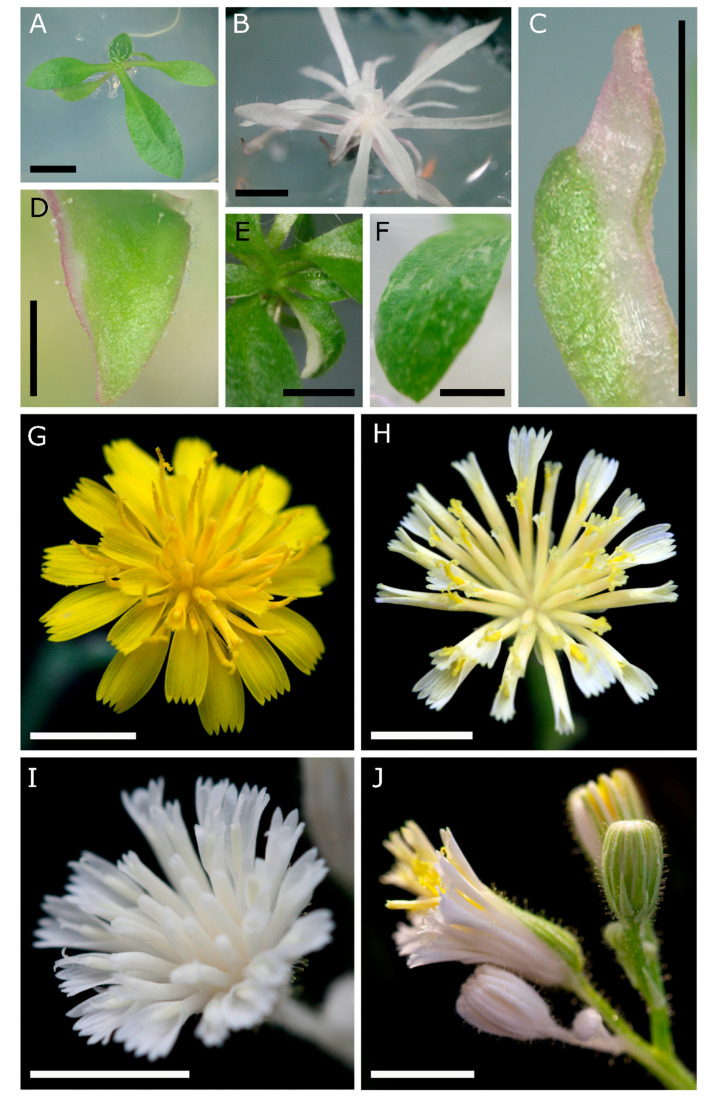
Vegetative and floral phenotypes of CRISPR/Cas9-induced *PDS* mutations in T0 *Hieracium* plants. (**A**) Non-edited transgenic plant with green shoots; (**B**) full albino plant; (**C**) sectorial chimera of a single emerging leaf with accumulation of pink anthocyanin pigments; (**D**) patch of albino cells on the edge of an expanding leaf; (**E**) chimeric leaf visible within a whole rosette; (**F**) leaf with variegated patchy albino appearance; (**G**) flower from a nontransgenic plant at anthesis; (**H**) flower from a chimeric *pds* transgenic at anthesis displaying pale-yellow to white petals within each floret, but normal yellow-colored bilobed stigmas; (**I**) full albino flower showing bleached florets, including white stigmas; (**J**) chimeric plant with both normal, sectorial and full albino capitula, visualized as white sepal coloration. Scale bar = 5 mm (**A**–**C**,**E**–**J**) or 1 mm (**D**).

**Figure 3:**
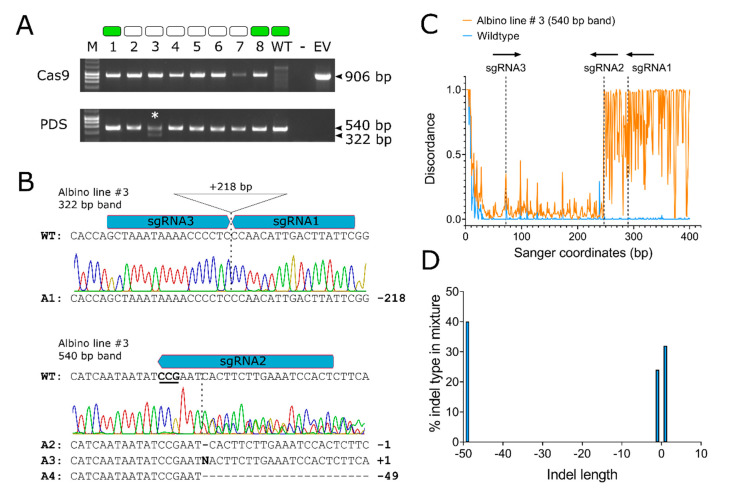
Detecting the presence of the transgene and CRISPR/Cas9-induced indels, by PCR and Sanger sequencing. (**A**) T-DNA integration was confirmed by PCR amplification of a 906 bp fragment of the *Streptococcus pyogenes Cas9* gene (upper gel). Edits were detected by PCR amplification of a 540 bp fragment of the *Hieracium PDS* gene (lower gel). Phenotypes of the plants that were examined in this assay are depicted above the gel as green (wildtype) or white (albino) boxes. The D36 wildtype control (WT), no template control (–) and pDE-Cas9 empty vector positive control (EV) are also shown. (**B**) Sanger sequencing chromatograms of the gel purified 340-bp band (upper) and 540-bp band (lower) shown in lane 3 (asterisk). The position of the sgRNAs within the wildtype (WT) sequence is shown above the chromatograms, PAM is underlined, and the predicted cut sites are shown as vertical dashed lines. Alleles inferred in the edited sequences are shown below the chromatograms; (**C**) visualization of the discordance between Sanger traces of the ~540-bp band derived from the wild type (blue) and albino line # 3 (orange), determined using ICE analysis. The expected cut sites for each sgRNA are shown as vertical dotted lines; (**D**) inferred alleles and their frequencies, detected within the ~540-bp band of albino line 3, by Synthego ICE analysis.

A total of 35 primary (T0) transgenic plants containing the construct targeting *HPDS* were generated from three independent experiments (Table 1; Figure 2). All transgenic lines tested positive by PCR for a 906 bp fragment corresponding to the *Cas9* gene (Figure 3A, upper panel). Four of the transgenic plants (11.4%) were completely albino showing the expected loss-of-function *pds* phenotype comprising loss of chlorophyll pigments (Table 1). Their rosette leaves were white and narrower relative to untransformed control seedlings (Figure 2A,B). Eleven T0 transgenics (31.4%) exhibited chimeric phenotypes with leaves that showed sectorial patterning (Figure 2C–E) and patchy loss of pigmentation (Figure 2F). In some cases, during early in vitro growth stages, albinism coincided with the accumulation of anthocyanin pigments (Figure 2B–D). The remaining twenty transgenic plants (57.1%) showed no obvious albino phenotype (Table 1).

Completely albino plants did not survive to develop flowers. Therefore, the chimeric transgenics were grown to the flowering stage, and the phenotypes within the capitula were assessed. Compared to untransformed D36 control capitula (Figure 2G), florets in capitula of chimeric transgenics displayed weak carotenoid pigmentation in the petals, possessing yellow pigmented stigmas (Figure 2H). In addition, fully albino composite flowers were observed (albeit less frequently) that showed a total loss of pigmentation in the sepals, petals, stamens and carpels (Figure 2I). Panicles of flowers in chimeric *HPDS*-edited plants contained a mixture of green and white capitula, as well as sectorially mutated capitula that displayed a mixture of white and green tissues (Figure 2J). These results indicate that CRISPR/Cas9-induced mutations in chimeric *Hieracium* transformants occurs in reproductive organs, which may include sporophytic and gametophytic cells.

### 3.2. CRIPSR/Cas9-Induced Indels and Deletions Can Be Rapidly Detected in Transgenic Hieracium Using PCR and Direct Sequencing

A rapid screening approach combining PCR amplification of the target region followed by Sanger sequencing and analysis was used to initially detect edits. The power of this approach is demonstrated here in a representative analysis of a putative tetra-allelic albino knockout (line #3). A 540 bp region of the *HPDS* gene targeted for mutagenesis by all three guide RNAs was amplified from the T0 transgenic lines (Figure 3A, lower panel). In albino line #3, two distinct bands were seen on the agarose gel (540 bp and 322 bp), suggesting that a deletion event had occurred (Figure 3A, lane 3). Sanger sequencing of the 322 bp amplicon revealed a clean chromatogram with single peaks, suggesting that the deletion had occurred in a single allele (Figure 3B). Alignment with the reference sequence showed that a 218 bp deletion had occurred precisely between the region 3 bp upstream of the PAMs in sgRNA1 and sgRNA3 (Figure 3B). Conversely, Sanger sequencing of the larger 540-bp band revealed regions with polymorphic sequence peaks commencing from within the sgRNA2-binding site, which indicated that this band consisted of multiple alleles (Figure 3B). Similar polymorphic sequence peaks were observed for other 540-bp bands (Figure S4). Analysis of the 540-bp chromatogram from Line #3 was performed using the Inference of CRISPR Edits (ICE) tool [38] (Figure 3C,D). Three alleles highly likely to contain indels (*R^2^* = 0.96) were found, including single base deletions and insertions, plus a 49 base deletion between guides sgRNA1 and sgRNA2 (Figure 3B,D). These data suggest that the albino Line #3 contained tetra-allelic disruptions, one of which was a 218 bp deletion between the two most distant sgRNAs. These analyses demonstrated that PCR in conjunction with Sanger sequencing can rapidly detect indels and infer editing outcomes in *Hieracium*. However, as all three guides were complementary to the target amplicon in this study, not all possible editing outcomes could be inferred using this method. A comprehensive assessment was, therefore, performed.

### 3.3. Amplicon Deep Sequencing Reveals a Wide Range of CRISPR/Cas9-Induced Indels in Hieracium Primary Transformants

**Figure 4:**
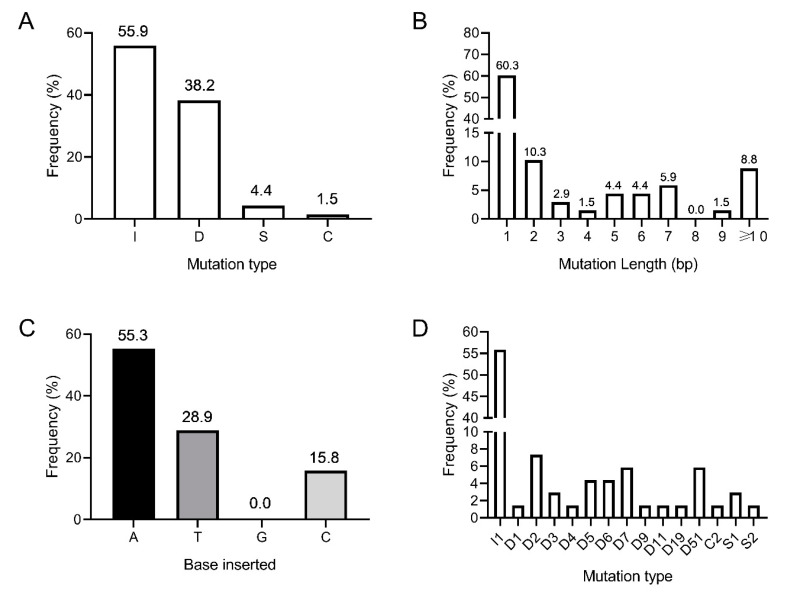
The combined types and frequencies of CRISPR/Cas9-induced mutations in the *Hieracium PDS* gene, mediated by the three different sgRNAs, determined by NGS in 18 T0 independent transformants. (**A**) Frequency of insertions [I], deletions [D], substitutions [S], and combined [C] mutation types; (**B**) Frequency of different mutation lengths regardless of the mutation types; (**C**) Percentage of bases inserted for the 1-bp insertions A = adenine, T = thymine, G = guanine, C = cytosine; (**D**) Frequency of each mutation type for all of the mutations induced by the three sgRNAs. I = insertion; D = deletion; S = substitution; C = combined mutation. Frequencies are shown above the bars in **A**–**C**.

To examine in more detail the type and frequency of CRISPR/Cas9-induced edits in the *HPDS* gene, amplicon deep sequencing was performed on 18 independent T0 transformants (6 green, 5 chimeric and 7 albino). The 7 albino lines included those shown in Table 1 and an additional three plants from leaf-disk transformations where phenotypic frequencies were not scored. Edits were identified as variations in the amplicon sequences near the predicted cleavage sites from those of an untransformed D36 control. Analysis of the combined mutations across two amplicons in 18 transgenic lines revealed differing frequencies of insertions (55.9%), deletions (38.2%), substitutions (4.4%) and combined mutations (1.5%) (i.e., >1 mutation type in an allele) (Figure 4A, Table S2). Single base mutations occurred 60.3% of the time (Figure 4B, Table S2). Adenine was the most frequently inserted single base, accounting for more than half of the 1 base insertions (55.3%), while single guanine insertions were not observed in our selection of transgenics (Figure 4C, Table S2). These single base insertion frequencies are similar to those reported in *Brassica napus* [39]. Promisingly, 6% of the editing events in regenerated *Hieracium* transgenics were large deletions between two distant guide RNAs (Figure 4D). The largest mutation that could be detected by NGS was a 51 bp deletion between the guides sgRNA1 and sgRNA2 (Figure 4D, Table S2). As the binding site of the third guide RNA (sgRNA3) was present within a separate amplicon, we did not detect the largest 218 bp deletions between the two most distant guides using the NGS method, but this was readily observed using standard PCR (Figure 3). Interestingly, three independently transformed green plants without a visible phenotype (Lines 4.1, 9.2 and 29.1) harbored CRISPR induced edits in *HPDS* (Table S2). These plants also retained wildtype *HPDS* alleles at relatively high frequencies (Table S2). This result confirms that complete knockout of all *HPDS* alleles is required to observe the albino phenotype in *Hieracium* cells.

### 3.4. Editing in Chimeric Hieracium Continues through Apomictic Seed Formation and Is Inherited in the Next Generation

The transmission of CRISPR/Cas9-mediated edits in seeds derived from the apomict T0 transgenics was examined. D36 is a self-incompatible facultative apomict. More than 97% of seeds set arise asexually via apomixis and are tetraploid maternal clones [40]. However, if the aposporous initial cell does not form in an ovule then meiosis can proceed forming a sexually derived female gametophyte where chromosomal segregation has occurred. Viable dihaploid (n + 0) seeds form if the *LOP* locus is inherited in meiotically reduced embryo sacs [41]. To test for *Cas9* segregation, 79 T1 progeny from two independent primary transformants (phenotypic chimeras) with confirmed edits were screened by PCR for the presence of the *Cas9*, showing that 98.7% (78/79) of seedlings contained the *Cas9* cassette (Figure S5). Segregation of *Cas9* in 1.3% of seedlings is consistent with a previous study where approximately 2.5% of D36 seeds were found to be meiotically derived [40]. Polyembryony occurred in 2% and 7% of the seed from the T0 *HPDS* lines 1 and 2, respectively (Table 2). These frequencies align with previous observations of polyembryony in D36, measured at embryo sac maturation [42] and germination [43]. These results suggest that most T1 seeds formed in the transgenic plants arose via apomixis.

**Figure 5:**
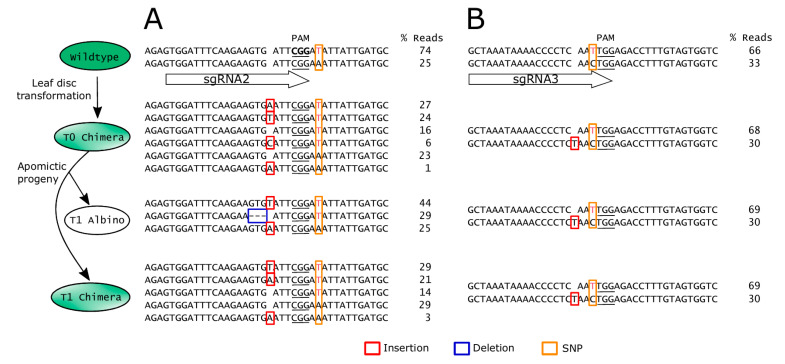
Stability of edits in the *HPDS* gene across different generations of apomictic *Hieracium* is affected by polymorphisms in the guide RNA. A subset of amplicon deep sequencing results surrounding the (**A**) sgRNA2 and (**B**) sgRNA3 binding sites. For clarity, sequences around the sgRNA3 binding site in (**B**) are shown as reverse complement. For each generation and each sgRNA target site, the allelic sequences from one representative plant are shown. Allele frequencies are shown as percentage of total reads. Note that sgRNA2 was 100% complementary to all alleles detected in the wildtype, but sgRNA3 was only 50% complementary due to a natural SNP 1 base upstream of the PAM. SNPs are shown in colored font with yellow boxes. Indels are shown as red boxes (insertion) or blue boxes (deletion). PAM underlined.

Two independent T0 chimeric parents produced a high percentage of full albino progeny at 62.9% and 84.6%, respectively, while the remaining T1 progeny exhibited albino sectors (Table 2). In *Arabidopsis*, chimeras can be eliminated from the T2 generation by self-fertilizing a chimeric T1 that contains edits in the germline [34]. However, D36 is a self-incompatible apomict, so the high frequency of albino seedlings observed in the T1 generation was likely due to continued activity of the Cas9 nuclease in the T0 parent and/or the parthenogenetic embryo. To test this hypothesis, *HPDS* gene edits were assessed across the T0 and T1 generations using NGS. Sequences from representative individual plants of each generation are illustrated in Figure 5. In the region surrounding sgRNA2, a T0 chimera contained edits in both wildtype alleles, but 39% of sequences remained unedited (Figure 5A). However, a T1 albino progeny derived from this parent contained edits in close to 100% of the amplicon sequence reads, which included a 3 base de novo deletion (Figure 5A). This suggests that the Cas9 remained active in the T0 before or during aposporous embryo sac formation or in the developing T1 embryo. Some alleles that were present in the T0 chimera were absent in the T1 progeny (Figure 5A). This may represent spatial differences in Cas9-mediated edits throughout cells of the chimeric T0 parent.

Analysis of editing across the generations also revealed allele-specific editing in *Hieracium*. Within one of the *HPDS* alleles, a cytosine/thymine single nucleotide polymorphism (SNP) was present 1 base upstream of the PAM of the sgRNA3 site (Figure 5B). Hence sgRNA3 was complementary to only half of the wildtype *HPDS* alleles (Figure 5B). No edits were mediated by sgRNA3 in the noncomplementary allele in any of the plants analyzed in either the T0 or T1 generations (Figure 5B, Table S2). The allele complementary to sgRNA3 contained a thymine insertion in the T0 (Figure 5B). The frequency of this edited allele remained stable across the T0 and T1 generations, suggesting that an early editing event may have occurred in the T0 (Figure 5B). Collectively, these results show that CRISPR/Cas9 is efficient in *Hieracium* and can be employed to target specific alleles.

## 4. Discussion

In this study, the combination of *Agrobacterium*-mediated leaf disk transformation together with CRISPR/Cas9-mediated disruption of the *HPDS* gene has shown high frequencies of putative tetra-allelic editing in the T1 generation of isolates examined (62.9% & 84.6%) and, in chimeric plants, albino sectoring continued through floral development. The absence of *Cas9* segregation (Figure S5) and continually active Cas9 activity through the events of apomixis where meiosis is suppressed and fertilization is not necessary to induce seed formation likely facilitated these good editing frequencies.

Recovery of fully albino mutants in the T0 generation occurred at an average frequency of 11.4%, with a maximum observed frequency of 16.7% in Experiment 2 (Table 1). The efficiency of CRISPR/Cas9-mediated editing of the *PDS* gene was variable in other species. For example, 63% of transgenic Cavendish banana plants displayed full albinism [32], while fully albino cassava mutants occurred at frequency of 21% for cultivar 60444 and 52% for cultivar TME 204 [30]. Differences in ploidy may explain these variable efficiencies, as the editing events in cassava (diploid) and Cavendish banana (triploid) would need to occur in fewer alleles to induce a phenotype than in *H. piloselloides* (tetraploid). It would be interesting to test the efficiency of CRISPR/Cas9 within dihaploid (D18) *Hieracium*. The type of explants used may also influence regeneration efficiencies. Like our study, leaf discs were used to generate transgenic apple plants that carried CRISPR/Cas9-induced mutations in PDS [44]. In that study, full and partial albino phenotypes occurred in 32% of regenerated T0 plantlets, which more closely resembles our combined frequency of 42% (Table 1). Different promoters, the total number of sgRNAs used and copy number of target genes, may also influence editing efficiencies.

Results presented here pave the way for the identification of key genes involved in apomixis in *Hieracium* using a genome editing strategy together with a seed screening approach used in a prior γ-mutagenesis screen to identify apomixis loci in *Hieracium* [12]. The *LOA* locus in both apomictic *H. praealtum* (R35) and *H piloselloides* (D36) resides on the long arm of a single hemizygous chromosome. The chromosomal location of the *LOP* locus in *H. praealtum* (R35) remains unknown, and the exact sizes of *LOA* and *LOP* loci are unclear. Gene sequences, SNPs, and molecular markers linked to both loci are known [15,19,22,33] and could be used to design allele-specific distant guide RNAs that initially cause large deletions of multiple gene candidates. Large chromosomal deletions of up to 245 Kb have been observed in rice [45]. In this study, 6% of the editing events in regenerated *Hieracium* transgenics were 51 bp deletions between two guides (Figure 4D), and larger deletions were detected by Sanger sequencing (Figure 3A,B). Future studies in *Hieracium* could therefore deploy pairs of sgRNAs that generate large (Kb) fragment deletions via the NHEJ repair pathway, which would enable the functional analysis of large *cis*-regulatory domains or gene clusters within apomixis loci.

**Figure 6:**
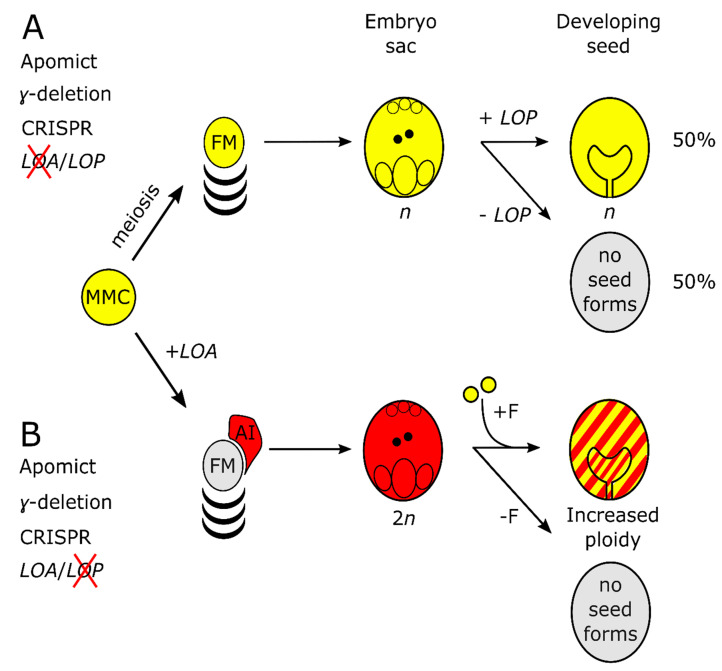
Schematic for identifying apomixis genes in *Hieracium* using functional knockouts and gene editing. (**A**) Known outcome of γ-mutagenesis and expected outcome of CRISPR-mediated disruption of *LOA* in apomictic tetraploid *Hieracium*. *LOA* loss-of-function causes *LOP* to segregate during meiosis. *LOP* activity in the reduced (n) embryo sacs leads to 50% dihaploid progeny and 50% non-viable seeds with aborted embryos. Gene edited progeny, from parents with reduced seed-set, could be screened by flow cytometry, sequencing and cytology; (**B**) γ deletions at *LOP* lead to formation of unreduced embryo sacs which, when fertilized (+F), increase in ploidy, hence the indicated red and yellow shading. Large and targeted CRISPR/Cas9 editing should result in a similar phenotype. Mutations in both loci would result in a sexual phenotype, as in Figure 1A.

The better characterized *LOA* locus could be a good initial focus for use of CRISPR/Cas9 to target multiple candidate apomixis genes simultaneously in this manner in *Hieracium*. *LOA* controls AI cell formation, sexual suppression and the formation of aposporous embryo sacs. If *LOA* were successfully deleted by CRISPR/Cas9, in tetraploid *H. piloselloides*, then the *LOP* locus would segregate in half of the progeny. Chromosomally reduced gametophytes containing *LOP* would develop into viable dihaploid (n + 0) seeds without fertilization (Figure 6A). If *LOP* were deleted, and *LOA* function remained intact, chromosomally unreduced gametophytes would require fertilization and the resultant progeny would show increased ploidy (Figure 6B). If both *LOA* and *LOP* were deleted, then seed formation would occur via sexual reproduction. These outcomes could be screened initially by seedling and ploidy phenotypes (Figure 6A,B) before progressing to a cytological and molecular assessment to identify functional knockouts in causal apomixis genes [12,46]. Allele specificity would avoid potentially lethal mutations in gene homeologs. A SNP was sufficient to confer allele-specific editing of the *CHLOROPLASTOS ALTERADOS1* (GhCLA1) gene in allotetraploid cotton (*Gossypium hirsutum*) [47]. Similarly, in our study, allele-specific editing was observed at the sgRNA3 target site due to a SNP (Figure 5B). Reversion to meiosis though the inactivation of *LOA* function would enable *Cas9* segregation in the progeny, which would facilitate rapid genetic screening. Candidate apomixis genes identified in this approach could then be transformed into characterized apomictic mutants deficient in *LOA* and *LOP* function to determine whether activity was restored [11].

## Figures and Tables

**Table 1:** Phenotypic frequency of recovered transformants after CRISPR/Cas9-mediated editing of the *Hieracium PDS* gene.

	Experiment 1		Experiment 2		Experiment 3		Combined Total	
	No. Plants	% Total	No. Plants	% Total	No. Plants	% Total	No. Plants	% Total
Green	9	64.3	6	50.0	5	55. 6	20	57.1
Albino	1	7.1	2	16.7	1	11.1	4	11.4
Chimera	4	28.6	4	33.3	3	33.3	11	31.4

**Table 2:** Phenotypes of T1 seedlings from two independent primary (T0) transformants. Seeds derived from chimeric plants.

	*HPDS* Line 1		*HPDS* Line 2	
	Progeny	%	Progeny	%
Albino	95	62.9	104	84.6
Chimera	56	37.1	19	15.4
Green	0	0	0	0
Polyembryony	2	1.3	7	5.7

## Supplementary Materials

The following are available online at https://www.mdpi.com/2073-4425/11/9/1064/s1. Table S1: List of primers used in the study. Table S2: Frequencies of edited *HPDS* alleles identified by next generation sequencing in T0 independent transformants. Figure S1: Schematic of the predicted *Hieracium* D18 *PHYTOENE DESATURASE* gene ortholog, guide RNA-binding sites and the CRISPR/Cas9 construct targeting the *HPDS* gene. Figure S2: Phylogenetic relationships of plant phytoene desaturase proteins. Figure S3: Functional testing of single guide RNAs by an in vitro cleavage assay. Figure S4: Sanger sequencing traces of the edited *HPDS* gene from multiple T0 replicate lines. Figure S5: Detection of the *Cas9* transgene in 78/79 T1 progeny from T0 chimeric *HPDS* mutants. References [48,49,50] are cited in the supplementary materials.

## References

[1] Conner J.A., Ozias-Akins P., Schmidt A. (2017). Apomixis: Engineering the ability to harness hybrid vigor in crop plants. Plant Germline Development. Methods in Molecular Biology.

[2] Hand M.L., Koltunow A.M. (2014). The genetic control of apomixis: Asexual seed formation. Genetics.

[3] Koltunow A.M., Grossniklaus U. (2003). Apomixis: A developmental perspective. Annu. Rev. Plant Biol..

[4] Sailer C., Schmid B., Grossniklaus U. (2016). Apomixis allows the transgenerational fixation of phenotypes in hybrid plants. Curr. Biol..

[5] Hörandl E., Temsch E.M. (2009). Introgression of apomixis into sexual species is inhibited by mentor effects and ploidy barriers in the Ranunculus auricomus complex. Ann. Bot..

[6] Conner J.A., Mookkan M., Huo H., Chae K., Ozias-Akins P. (2015). A parthenogenesis gene of apomict origin elicits embryo formation from unfertilized eggs in a sexual plant. Proc. Natl. Acad. Sci. USA.

[7] Bortesi L., Fischer R. (2015). The CRISPR/Cas9 system for plant genome editing and beyond. Biotechnol. Adv..

[8] Jia H., Wang N. (2014). Targeted genome editing of sweet orange using Cas9/sgRNA. PLoS ONE.

[9] Jia H., Zhang Y., Orbović V., Xu J., White F.F., Jones J.B., Wang N. (2017). Genome editing of the disease susceptibility gene CsLOB1 in citrus confers resistance to citrus canker. Plant Biotechnol. J..

[10] Hand M.L., Vit P., Krahulcova A., Johnson S.D., Oelkers K., Siddons H., Chrtek J., Fehrer J., Koltunow A.M.G. (2015). Evolution of apomixis loci in Pilosella and Hieracium (Asteraceae) inferred from the conservation of apomixis-linked markers in natural and experimental populations. Heredity.

[11] Koltunow A.M.G., Johnson S.D., Rodrigues J.C.M., Okada T., Hu Y., Tsuchiya T., Wilson S., Fletcher P., Ito K., Suzuki G. (2011). Sexual reproduction is the default mode in apomictic *Hieracium* subgenus *Pilosella*, in which two dominant loci function to enable apomixis. Plant J..

[12] Catanach A.S., Erasmuson S.K., Podivinsky E., Jordan B.R., Bicknell R. (2006). Deletion mapping of genetic regions associated with apomixis in *Hieracium*. Proc. Natl. Acad. Sci. USA.

[13] Henderson S.T., Johnson S.D., Eichmann J., Koltunow A.M.G. (2017). Genetic analyses of the inheritance and expressivity of autonomous endosperm formation in Hieracium with different modes of embryo sac and seed formation. Ann. Bot..

[14] Ogawa D., Johnson S.D., Henderson S.T., Koltunow A.M. (2013). Genetic separation of autonomous endosperm formation (AutE) from the two other components of apomixis in Hieracium. Plant Reprod..

[15] Okada T., Ito K., Johnson S.D., Oelkers K., Suzuki G., Houben A., Mukai Y., Koltunow A.M. (2011). Chromosomes carrying meiotic avoidance loci in three apomictic eudicot *Hieracium* subgenus *Pilosella* species share structural features with two monocot apomicts. Plant Physiol..

[16] Akiyama Y., Conner J.A., Goel S., Morishige D.T., Mullet J.E., Hanna W.W., Ozias-Akins P. (2004). High-resolution physical mapping in *Pennisetum squamulatum* reveals extensive chromosomal heteromorphism of the genomic region associated with apomixis. Plant Physiol..

[17] Akiyama Y., Hanna W.W., Ozias-Akins P. (2005). High-resolution physical mapping reveals that the apospory-specific genomic region (ASGR) in Cenchrus ciliaris is located on a heterochromatic and hemizygous region of a single chromosome. Theor. Appl. Genet..

[18] Bräuning S., Catanach A., Lord J.M., Bicknell R., Macknight R.C. (2018). Comparative transcriptome analysis of the wild-type model apomict *Hieracium praealtum* and its *loss of parthenogenesis* (*lop*) mutant. BMC Plant Biol..

[19] Rabiger D.S., Taylor J.M., Spriggs A., Hand M.L., Henderson S.T., Johnson S.D., Oelkers K., Hrmova M., Saito K., Suzuki G. (2016). Generation of an integrated *Hieracium* genomic and transcriptomic resource enables exploration of small RNA pathways during apomixis initiation. BMC Biol..

[20] Juranić M., Tucker M.R., Schultz C.J., Shirley N.J., Taylor J.M., Spriggs A., Johnson S.D., Bulone V., Koltunow A.M. (2018). Asexual female gametogenesis involves contact with a sexually-fated megaspore in apomictic *Hieracium*. Plant Physiol..

[21] Okada T., Hu Y., Tucker M.R., Taylor J.M., Johnson S.D., Spriggs A., Tsuchiya T., Oelkers K., Rodrigues J.C.M., Koltunow A.M.G. (2013). Enlarging cells initiating apomixis in *Hieracium praealtum* transition to an embryo sac program prior to entering mitosis. Plant Physiol..

[22] Kotani Y., Henderson S.T., Suzuki G., Johnson S.D., Okada T., Siddons H., Mukai Y., Koltunow A.M.G. (2014). The *LOSS OF APOMEIOSIS* (*LOA*) locus in *Hieracium praealtum* can function independently of the associated large-scale repetitive chromosomal structure. New Phytol..

[23] Bicknell R.A., Borst N.K. (1994). Agrobacterium-mediated transformation of *Hieracium aurantiacum*. Int. J. Plant Sci..

[24] Bertier L.D., Ron M., Huo H., Bradford K.J., Britt A.B., Michelmore R.W. (2018). High-resolution analysis of the efficiency, heritability, and editing outcomes of CRISPR/Cas9-induced modifications of *NCED4* in lettuce (*Lactuca sativa*). G3: Genes|Genomes|Genet..

[25] Shan S., Mavrodiev E.V., Li R., Zhang Z., Hauser B.A., Soltis P.S., Soltis D.E., Yang B. (2018). Application of CRISPR/Cas9 to *Tragopogon* (Asteraceae), an evolutionary model for the study of polyploidy. Mol. Ecol. Resour..

[26] Kishi-Kaboshi M., Aida R., Sasaki K. (2017). Generation of gene-edited *Chrysanthemum morifolium* using multicopy transgenes as targets and markers. Plant Cell Physiol..

[27] Iaffaldano B., Zhang Y., Cornish K. (2016). CRISPR/Cas9 genome editing of rubber producing dandelion *Taraxacum kok-saghyz* using *Agrobacterium rhizogenes* without selection. Ind. Crop. Prod..

[28] Qin G., Gu H., Ma L., Peng Y., Deng X.W., Chen Z., Qu L.J. (2007). Disruption of phytoene desaturase gene results in albino and dwarf phenotypes in Arabidopsis by impairing chlorophyll, carotenoid, and gibberellin biosynthesis. Cell Res..

[29] Zhang H., Zhang J., Wei P., Zhang B., Gou F., Feng Z., Mao Y., Yang L., Zhang H., Xu N. (2014). The CRISPR/Cas9 system produces specific and homozygous targeted gene editing in rice in one generation. Plant Biotechnol. J..

[30] Odipio J., Alicai T., Ingelbrecht I., Nusinow D.A., Bart R., Taylor N.J. (2017). Efficient CRISPR/Cas9 genome editing of phytoene desaturase in cassava. Front. Plant Sci..

[31] Howells R.M., Craze M., Bowden S., Wallington E.J. (2018). Efficient generation of stable, heritable gene edits in wheat using CRISPR/Cas9. BMC Plant Biol..

[32] Naim F., Dugdale B., Kleidon J., Brinin A., Shand K., Waterhouse P., Dale J. (2018). Gene editing the phytoene desaturase alleles of Cavendish banana using CRISPR/Cas9. Transgenic Res..

[33] Shirasawa K., Hand M.L., Henderson S.T., Okada T., Johnson S.D., Taylor J.M., Spriggs A., Siddons H., Hirakawa H., Isobe S. (2015). A reference genetic linkage map of apomictic Hieracium species based on expressed markers derived from developing ovule transcripts. Ann. Bot..

[34] Fauser F., Schiml S., Puchta H. (2014). Both CRISPR/Cas-based nucleases and nickases can be used efficiently for genome engineering in *Arab*. Thaliana Plant J..

[35] Pinello L., Canver M.C., Hoban M.D., Orkin S.H., Kohn D.B., Bauer D.E., Yuan G.-C. (2016). Analyzing CRISPR genome-editing experiments with CRISPResso. Nat. Biotechnol..

[36] Clement K., Rees H., Canver M.C., Gehrke J.M., Farouni R., Hsu J.Y., Cole M.A., Liu D.R., Joung J.K., Bauer D.E. (2019). CRISPResso2 provides accurate and rapid genome editing sequence analysis. Nat. Biotechnol..

[37] Edwards K., Johnstone C., Thompson C. (1991). A simple and rapid method for the preparation of plant genomic DNA for PCR analysis. Nucleic Acids Res..

[38] Hsiau T., Maures T., Waite K., Yang J., Kelso R., Holden K., Stoner R. (2018). Inference of CRISPR edits from Sanger trace data. bioRxiv.

[39] Yang H., Wu J.-J., Tang T., Liu K.-D., Dai C. (2017). CRISPR/Cas9-mediated genome editing efficiently creates specific mutations at multiple loci using one sgRNA in *Brassica napus*. Sci. Rep..

[40] Bicknell R.A., Lambie S.C., Butler R.C. (2003). Quantification of progeny classes in two facultatively apomictic accessions of *Hieracium*. Hereditas.

[41] Bicknell R.A., Koltunow A.M. (2004). Understanding apomixis: Recent advances and remaining conundrums. Plant Cell.

[42] Juranić M., Johnson S.D., Koltunow A.M. (2019). Phenotypic plasticity of aposporous embryo sac development in *Hieracium praealtum*. Plant Signal. Behav..

[43] Koltunow A.M., Johnson S.D., Bicknell R.A. (2000). Apomixis is not developmentally conserved in related, genetically characterized *Hieracium* plants of varying ploidy. Sex Plant Reprod..

[44] Nishitani C., Hirai N., Komori S., Wada M., Okada K., Osakabe K., Yamamoto T., Osakabe Y. (2016). Efficient genome editing in apple using a CRISPR/Cas9 system. Sci. Rep..

[45] Zhou H., Liu B., Weeks D.P., Spalding M.H., Yang B. (2014). Large chromosomal deletions and heritable small genetic changes induced by CRISPR/Cas9 in rice. Nucleic Acids Res..

[46] Koltunow A.M.G., Johnson S.D., Okada T. (2011). Apomixis in hawkweed: Mendel’s experimental nemesis. J. Exp. Bot..

[47] Wang P., Zhang J., Sun L., Ma Y., Xu J., Liang S., Deng J., Tan J., Zhang Q., Tu L. (2018). High efficient multisites genome editing in allotetraploid cotton (*Gossypium hirsutum*) using CRISPR/Cas9 system. Plant Biotechnol. J..

[48] Larkin M.A., Blackshields G., Brown N.P., Chenna R., McGettigan P.A., McWilliam H., Valentin F., Wallace I.M., Wilm A., Lopez R. (2007). Clustal W and Clustal X version 2.0. Bioinformatics.

[49] Shan S., Mavrodiev E.V., Li R., Zhang Z., Hauser B.A., Soltis P.S., Soltis D.E., Yang B. (2018). Data from: Application of CRISPR/Cas9 to Tragopogon (Asteraceae), an evolutionary model for the study of polyploidy. Dryad Digit. Repository.

[50] Kumar S., Steche G., Li M., Knyaz C., Tamura K. (2018). MEGA X: Molecular evolutionary genetics analysis across computing platforms. Mol. Biol. Evol..

