# Supplementary material for: Efficient CRISPR/Cas9-Mediated Knockout of an Endogenous PHYTOENE DESATURASE Gene in T1 Progeny of Apomictic Hieracium Enables New Strategies for Apomixis Gene Identification
Source: Genes (Basel). 2020 Sep 10;11(9):1064. doi: 10.3390/genes11091064 (PMC7563859; doi:10.3390/genes11091064)
Supplement: Supplementary file 1 [file genes-11-01064-s001.zip › Henderson_et_al_Genes_Supp_Resubmission.docx]

**
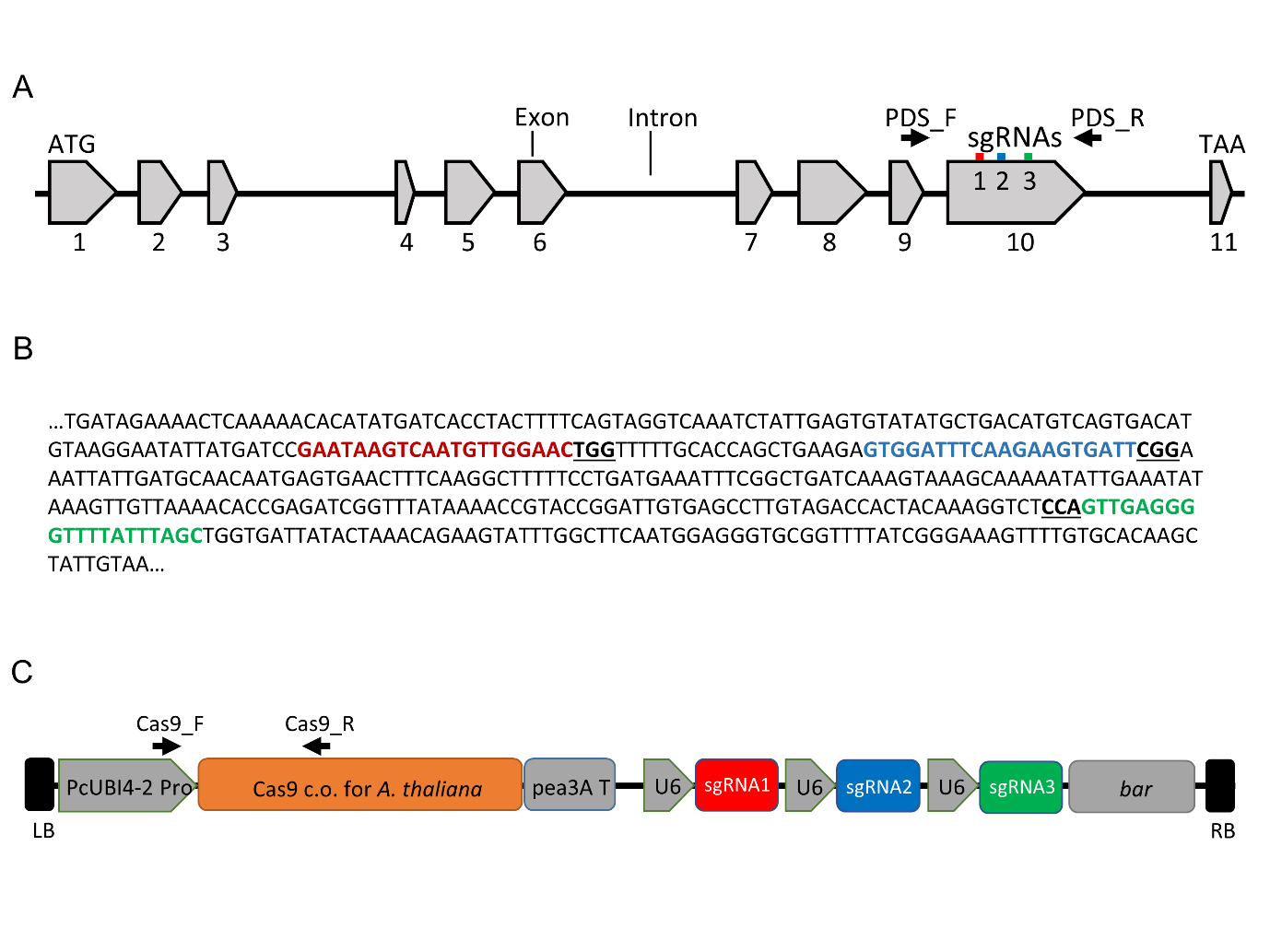
Figure S1: Schematic of the predicted *Hieracium* D18 *PHYTOENE DESATURASE* (*HPDS*) gene ortholog, guide RNA binding sites, and the CRISPR/Cas9 construct targeting the *HPDS* gene.** (A) Structural organisation of the *HPDS* gene. Three single guide RNAs (sgRNAs) were designed from exon 10. (B) Sequence of exon 10 showing the location of three 20 bp sgRNAs. PAM underlined. (C) The CRISPR/Cas9 binary vector pDE-Cas9 used for stable Agrobacterium-mediated transformation of *Hieracium*. The parsley Ubiquitin 4-2 promoter (PcUbi4-2 pro) drives *Cas9* expression. *Arabidopsis thaliana* AtU6-26 promoters (U6) drive the sgRNAs. Forward and reverse primers used to screen plants by PCR (in Fig. 3A) are depicted as arrows.


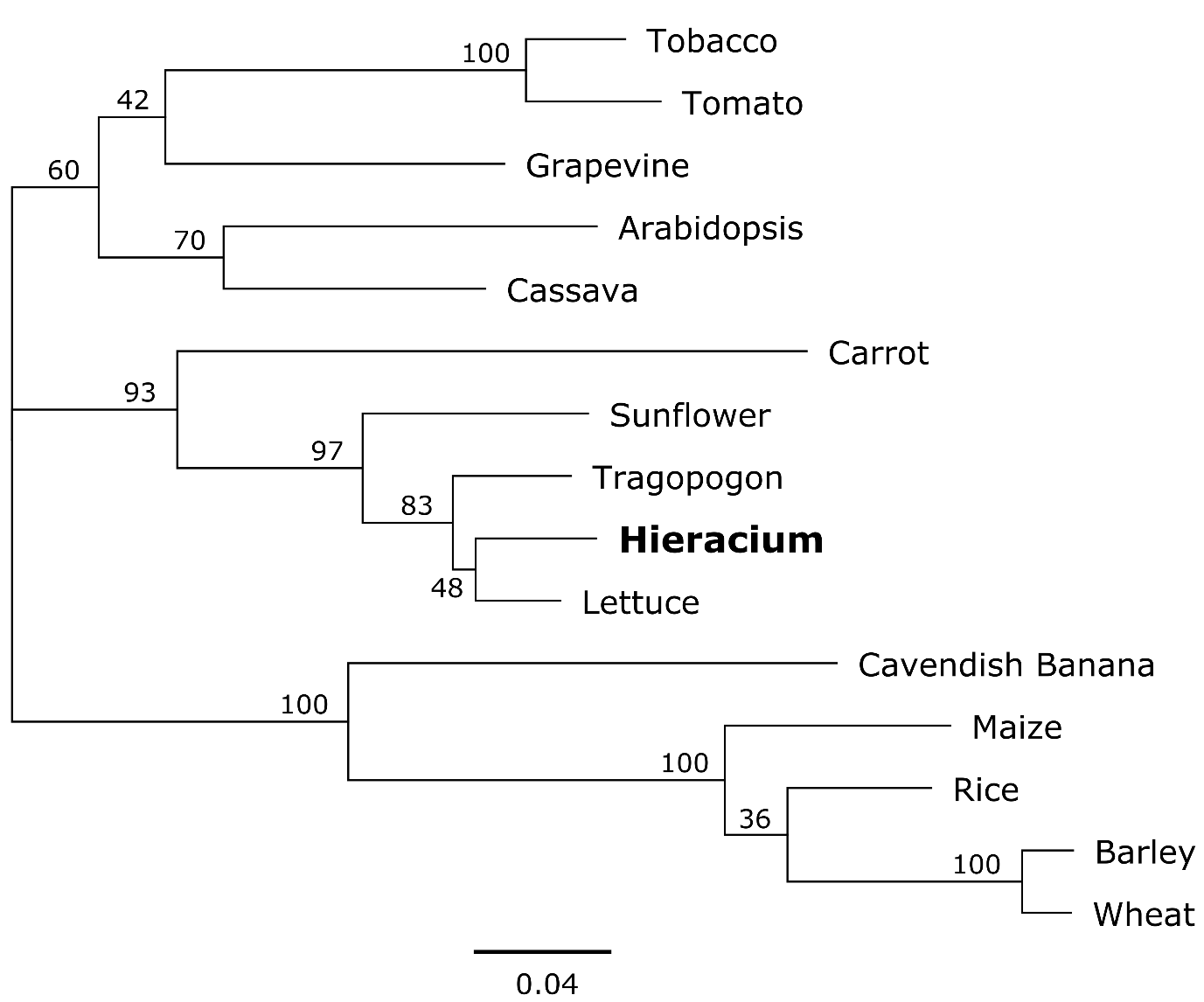


**Figure S2: Phylogenetic relationships of plant Phytoene Desaturase proteins.** A Maximum Likelihood phylogenetic tree was inferred from an amino acid sequence alignment generated using ClustalW [48]. Bootstrap values from 1000 iterations are shown next to the branches. Tree with the highest log likelihood (-6441.19) is shown. The NCBI accession numbers that were used are: Arabidopsis (*Arabidopsis thaliana*) [L16237.1], Lettuce (*Lactuca sativa*) [PLY83262], Grapevine (*Vitis vinifera*) [JQ319635.1], Tomato (*Solanum lycopersicum*) [KC767848.1], Tobacco (*Nicotiana attenuate*) [JX185751.1], Carrot (*Daucus carota*) [NM_001329175.1], Sunflower (*Helianthus annuus*) [KF263656.1], Cavendish banana (*Musa acuminata*) [JQ762260], Rice (*Oryza sativa*) [AF049356], Wheat (*Triticum aetivum*) [FJ517553], Maize (*Zea mays*) [U37285], Barley (*Hordeum vulgare*) [AK371906.1], Tragopogon (*Tragopogon dubius*) [49], Cassava (*Manihot esculenta*) [XP_021613095.1], and *Hieracium* (*Hieracium piloselloides*, diploid accession D18, 2x=2n=18) [19]. Evolutionary analyses were conducted in MEGA X [50]. Scale = number of substitutions per site.

19. Rabiger, D.S., et al., *Generation of an integrated Hieracium genomic and transcriptomic resource enables exploration of small RNA pathways during apomixis initiation.* BMC Biology, 2016. **14**(1): p. 86.

48. Larkin, M.A., et al., *Clustal W and Clustal X version 2.0.* Bioinformatics, 2007. **23**(21): p. 2947-2948.

49. Shan, S., et al., *Data from: Application of CRISPR/Cas9 to Tragopogon (Asteraceae), an evolutionary model for the study of polyploidy*. 2018, Dryad Digital Repository.

50. Kumar, S., et al., *MEGA X: molecular evolutionary genetics analysis across computing platforms.* Molecular Biology and Evolution, 2018. **35**(6): p. 1547-1549.

sgRNA

uncut

2

3

1


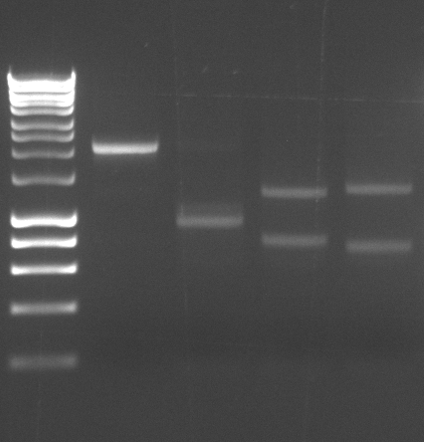


**Figure S3: Functional testing of single guide RNAs by an *in vitro* cleavage assay.**  All three sgRNAs used in this study were functional *in vitro*. A 2.17 kb PCR-amplified fragment of the *Hieracium PDS* gene was incubated without (lane 1, uncut), or with recombinant Cas9 protein and *in vitro* synthesised sgRNA1, sgRNA2, or sgRNA3. The cleaved fragments were separated on an agarose gel.

**
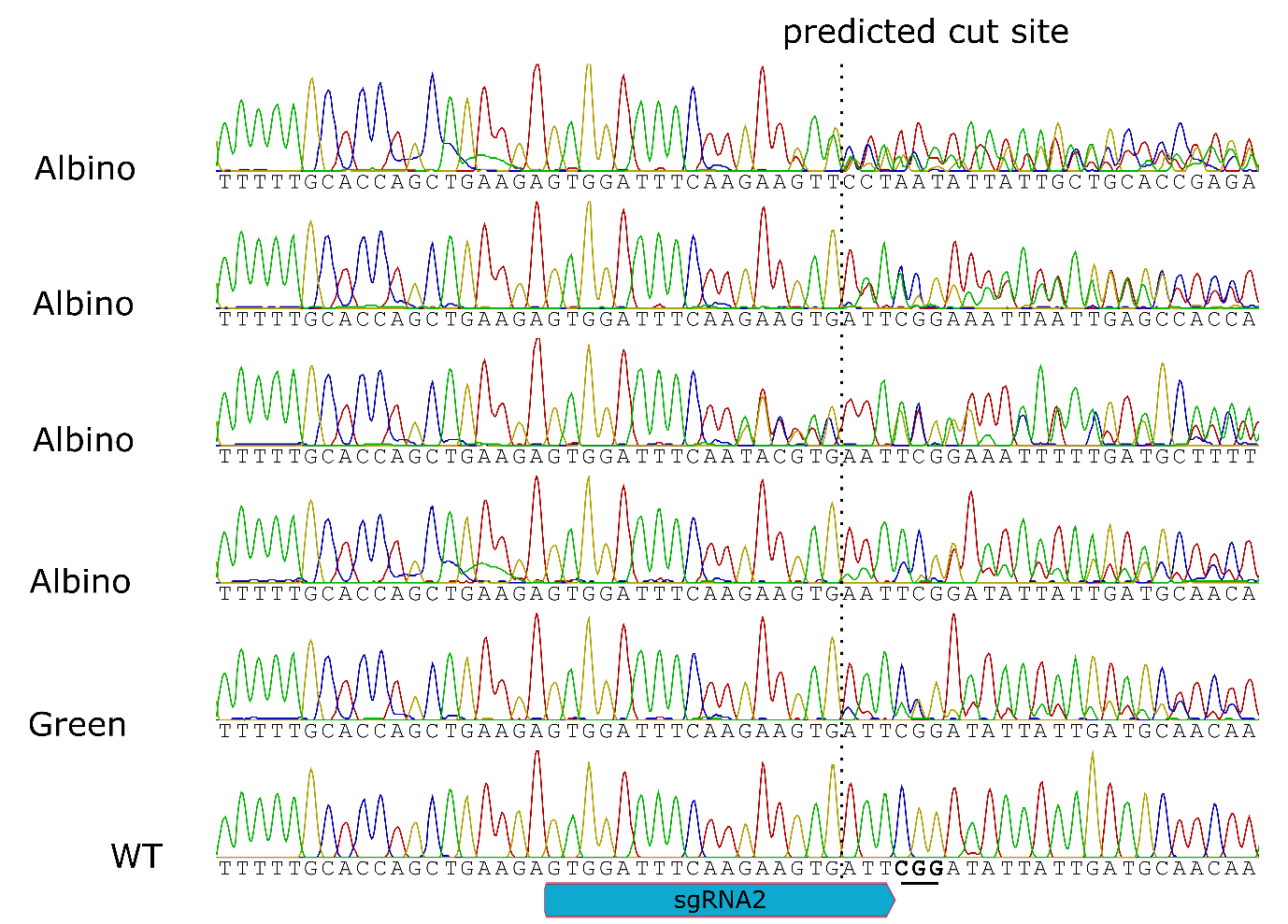
**

**Figure S4:** Sanger sequencing traces of the edited *HPDS* gene from multiple T0 replicate lines.

Chromatogram traces represent a 540 bp DNA fragment of *HPDS* generated by PCR, and sequenced from five independent T0 transformants. The phenotypes (albino or green) are indicated at the left of the traces. A wildtype plant (WT) was sequenced as a control. The predicted cut site is shown as a vertical dashed line. The sgRNA2 binding site is shown below the traces. PAM underlined.

**
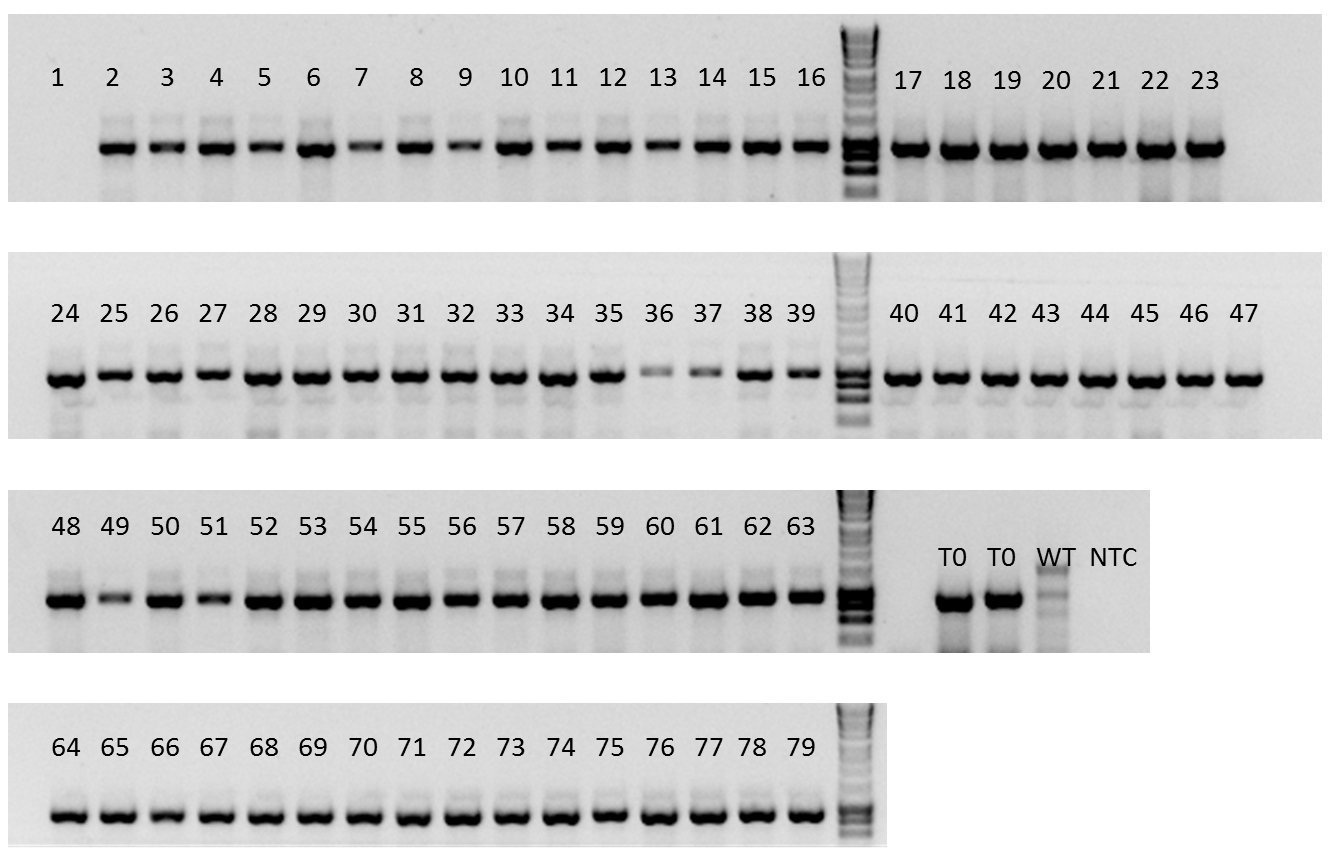
**

**Figure S5: Detection of the *Cas9* transgene in 78/79 T1 progeny from T0 chimeric *HPDS* mutants.** The *Cas9* cassette was amplified from whole seedling genomic DNA by PCR using primers SWH190 and SWH191 (Table S1). The 906 bp fragments were separated on an agarose gel. T0 = parent plant; WT = untransformed *H. piloselloides* line D36, NTC = no template control.
